# The added value of an internet-based intervention for treatment of aggression in forensic psychiatric outpatients—study protocol for a multicentre, mixed-methods randomized controlled trial

**DOI:** 10.1177/20552076241303835

**Published:** 2024-12-12

**Authors:** Hanneke Kip, Lisa Klein Haneveld, Saskia M Kelders

**Affiliations:** 1 Department of Psychology, Health & Technology, Centre for eHealth and Wellbeing Research, 3230 University of Twente, Enschede, The Netherlands; 2 Department of Research, Stichting Transfore, Deventer, The Netherlands; 3 Optentia Research Focus Area, North-West University, Vanderbijlpark, South Africa

**Keywords:** clinical trials, digital health, eHealth, mental health, mixed methods, psychology, psychology, studies

## Abstract

**Background:**

Even though internet-based interventions have been used in treatment of forensic psychiatric outpatients for over 10 years, no robust effectiveness studies have been conducted in this complex branch of mental healthcare.

**Objectives:**

To present the protocol of a study that investigates whether the addition of the internet-based intervention “Dealing with Aggression” to treatment as usual (TAU) leads to better treatment outcomes than TAU that is delivered solely in-person.

**Methods:**

This study uses a multicentre mixed-methods randomized controlled trial (RCT) design, with four Dutch forensic outpatient organizations. Patients are included if they receive outpatient treatment for aggression. They will be randomized into an experimental condition, in which the intervention is added to TAU (n = 64), or a control condition, with only TAU (n = 64). Participants are assessed four times: at baseline (T0), halfway during the 10-week intervention (T1), after completing the intervention (T2), and after 3 months (T3). Primary outcome measure is self-reported aggression, other outcome measures are regulatory emotional self-efficacy and treatment readiness, the number of treatment sessions, and dynamic risk factors. Adherence to and engagement with the internet-based intervention will be investigated as predictors for effectiveness. Perceived effect and points of improvement are identified via semistructured interviews with patients and therapists.

**Discussion:**

This will be the first study to investigate the effectiveness of an internet-based intervention in a forensic psychiatric outpatient sample. By means of the mixed-methods design and use of adherence and engagement as predictors, this study will answers questions about if, but also why and for whom this intervention works.

## Introduction

### Forensic mental healthcare

Forensic mental healthcare is a complex branch of care that takes place at the intersection of psychiatry and law. It is focused on treatment of patients that show aggressive or sexual delinquent behavior, are on the verge on or have committed a crime, and have one or more concurrent psychiatric disorders.^1^ Treatment takes place in inpatient and outpatient clinics and is often part of a sentence. There is not one “typical” forensic psychiatric patient: the population is very heterogenous in terms of psychiatric disorders, committed offense, and psychosocial problems.^2^ This heterogeneity, combined with generally low treatment motivation and low literacy levels,^3^ implies that this population can often be more complex to involve in treatment compared to nonforensic populations. To illustrate: research has shown that when looking at the use of cognitive behavioral therapy (CBT), effectiveness rates for aggression are lower than those for treatment of for example anxiety or mood disorders.^4,5^ Additionally, a meta-analysis on the efficacy of different types of psychological treatment in violent offenders showed that 50% of offenders who did not receive treatment would reoffend, as opposed to 38.8% of offenders who received treatment.^6^ This shows that, while treatment can be of added value, there is room for improvement. On top of this, there is a shortage of healthcare providers, while there also is an increase in forensic psychiatric patients that require treatment.^7^ Consequently, there is a need for novel approaches to make forensic mental healthcare more effective and efficient.

### Research on internet-based interventions in mental healthcare

Internet-based interventions are viewed as a promising way to improve effectiveness and efficiency of mental healthcare in general.^8^ These types of interventions are quite heterogeneous, since they differ in addressed outcomes, content, way of delivery (e.g. computer or smartphone), multimedia components (e.g. videos, written assignments, informative texts), and theoretical bases.^9^ In mental healthcare, many internet-based interventions are grounded in principles or elements of CBT. Furthermore, in clinical practice, these interventions are often used in a blended way, meaning that interventions are discussed during treatment sessions and patients often receive feedback from their therapist within the guided intervention.^10^ Meta-analyses consistently show that internet-based interventions are effective for different psychiatric disorders, such as depression or post-traumatic stress disorders.^11–15^ Additionally, research shows that CBT-based internet-based interventions yield similar effects as face-to-face therapy for different types of psychiatric disorders.^16^ However, these relatively positive findings on internet-based interventions in mental healthcare in general cannot simply be generalized to forensic practice. Forensic treatment is unique due to its legal component, its very complex treatment population with diverse psychosocial problems, and its main focus on preventing recidivism instead of on one type of psychiatric disorder.

### Research on internet-based interventions in forensic mental healthcare

While the potential of internet-based interventions for forensic treatment is widely recognized, not much is known about their effectiveness.^17^ A systematic review on eHealth in treatment of offenders identified only 13 (quasi-)experimental evaluation studies on internet-based interventions in treatment of offenders.^18^ Of these studies, 10 showed that internet-based interventions were at least as effective as standard care or more effective than waiting lists, while three found no evidence for effectiveness. While these initial findings are in line with meta-analyses on internet-based interventions in general mental healthcare, no reliable conclusions on effectiveness can be drawn due to the low number of studies and heterogeneity of settings in which they were conducted. Additionally, most studied interventions were not grounded in CBT and were not integrated in treatment in a blended way—even though this is viewed as the most optimal way to deliver internet-based interventions in forensic settings.^19^ Furthermore, outpatient settings were underrepresented, despite the potential that interventions have for these patients, especially considering the limited treatment time that they have. Qualitative research has shown that therapists believe that blended, CBT-based internet-based interventions can improve the effectiveness and efficiency of outpatient treatment in multiple ways.^20–23^ Among other things, they can provide the patient with more tools for behavior change in ways that fit their skills, such as explanatory videos or stories of peers. Additionally, because patients work on interventions independently and outside of treatment sessions, they might attribute improvement to their own activities than merely to the therapist, which can result in more self-efficacy and motivation to change. These interventions might also be able to increase the efficiency of care, for example by replacing parts of treatment that are usually delivered by therapists, for example, psycho-education. This can also allow patients to receive a higher dose of treatment without requiring more time with a therapist, which is especially relevant due to the shortages in staff.^24^

Most advantages of internet-based interventions for forensic patients are derived from qualitative research, highlighting the need for more robust effectiveness research in practice.^25^ Many therapists indicate that internet-based interventions do not fit the skills and needs of all forensic patients.^20^ This highlights the need to determine not just if, but also why internet-based interventions works for forensic psychiatric patients.^17^ Nonspecific predictors such as adherence and engagement offer possible answers to these questions, but they have not been studied in forensic settings yet.^26–30^ Additionally, to further unravel why an intervention does (not) work, a mixed-methods approach in which qualitative research is used to explain outcomes of quantitative results is recommended—especially when not much is known yet about effectiveness.^31^ Consequently, there is a need for more research on if and why internet-based interventions work in forensic psychiatric outpatient care.

### Research objectives

To evaluate the existing, CBT-based Dutch internet-based intervention “Dealing with Aggression” [Omgaan met Agressie], a mixed-methods randomized controlled trial (RCT) will be conducted. This intervention has already been used in blended form in Dutch clinical practice for over 10 years, but has not been evaluated yet. The main goal of this study is to investigate whether the addition of the intervention “Dealing with Aggression” to forensic psychiatric outpatient treatment as usual (TAU) results in better treatment outcomes (aggression, regulatory emotional self-efficacy, and treatment readiness), compared to patients that only receive in-person TAU. Furthermore, it is assessed if the addition of “Dealing with Aggression” leads to less in-person treatment session during the data collection period. Another objective is to investigate if adherence to and engagement with the intervention are predictors of effectiveness on the outcome measures. Additionally, the findings will be further explained by means of interviews with patients in the experimental group and their therapists.

## Methods

### Design

In this two-armed RCT, patients will be randomly allocated to an experimental group in which “Dealing with Aggression” is added to in-person TAU, or a control group that only receives TAU. The two conditions are compared at four points in time: directly before the start of using the internet-based intervention (T0), halfway through the intervention (T1), directly after completing the intervention (T2), and at follow up, 3 months after completing the intervention (T3). Adherence and engagement will be included as potential predictors of effectiveness, and qualitative data will be collected via semistructured interviews with participating patients and therapists. In this multisite study, participants from four different organizations will be included. To increase feasibility, the study is coordinated and monitored by a multidisciplinary project team with at least one representative from each participating organization. The team consists of researchers on eHealth and/or forensic psychiatry, clinical psychologists, managers, and a member of Minddistrict—the platform on which the intervention is delivered. This study was approved by the medical ethical board Oost-Nederland of the Radboud University Medical Centre (number NL80846.091.22) and will be conducted in accordance with the principles of the Declaration of Helsinki. The study is registered at ClinicalTrials.gov, number NCT05711342.

### Participants

All participants of this study are treated in a forensic psychiatric outpatient clinic, with one of their main treatment goals being focused on aggression regulation problems. Four Dutch forensic organizations that offer outpatient care participate: Transfore (four outpatient clinics), Kairos (two outpatient clinics), de Woenselse Poort (one outpatient clinic), and GGZ Noord-Holland-Noord (three outpatient clinics). Within these outpatient clinics, some patients receive treatment as part of their sentence, while others receive treatment on a voluntary basis. Because patients reside at home, therapists are not involved in any decisions about their sentence or leave.

In order to be eligible for participants, a patient must be 18 years or older, receive in-person one-on-one and/or group treatment focused on aggression, and the responsible therapist must indicate that participating is not expected to result in any harm. Patients are excluded if they are unable to read the Dutch language, currently reside in any psychiatric inpatient clinic, are in current psychiatric crisis, or if the responsible therapist identifies any other valid reason for exclusion. Because forensic psychiatric treatment and the intervention are both transdiagnostic, the target group is not specified by psychiatric disorders.

#### Recruitment

Therapists will screen patients on suitability for participation when their cases are first discussed in a “multidisciplinary consultation,” which takes place before treatment starts. After start of treatment, the therapists will wait 8 weeks before introducing the study to the patient and asks for their consent to be contacted by a researcher. In line with recommendations from therapists, reasons for this are to prevent that the patient feels too overwhelmed at the start of treatment, and to ensure that there is time to work on their therapeutic relationship before the internet-based intervention is introduced. The therapist will make clear that the decision to participate in the study will not influence their treatment progress in any way to prevent a possible experienced coercion within patients.

If the patient agrees to be contacted by a researcher, communication will take place via a method of their choice, for example, phone, videoconferencing, or in-person. The researcher will inform them verbally and via a tailored information folder about participation. It will be made clear to patients that if they are assigned to the control condition, they can still use any internet-based intervention after data collection is completed. Furthermore, the researcher will clearly explain that the decision to participate or drop out of the study will not affect their treatment progress in any way. After this, the patient has a week to consider participation. If they decide to do so, they are asked to sign an informed consent form.

#### Power and sample size calculation

No other studies on the evaluation of internet-based interventions in forensic outpatient care on which an effect size could be estimated were identified. Consequently, a systematic review on internet-delivered CBT for depression and anxiety disorders was used in which a mean effect size, as measured by Cohen's d, of .8 at follow-up after 3 to 36 months was found when looking at the post-test difference between treatment and control conditions.^11^ However, in forensic mental healthcare, lower effect sizes are expected due to the complexity of improving treatment outcomes, so the more moderate effect size (Cohen's d) of this RCT is set on .5 on the primary outcome at follow up (T3). A power analysis was conducted with G*power, with an effect size of .5, a β-power of .8, a p-value of 5%, and an independent two-sided t-test as method of analysis. This analysis showed that 64 patients need to be included in each condition, resulting in a total of 128 patients. Accounting for drop-out of 20%, the goal for inclusion is set at a total of 154 outpatients. Participants are considered as a “drop-out” when they don’t complete the questionnaires in more than one measuring moment; not when they stop using the intervention. The main reasons for this are that adherence will be used as a predictor for effectiveness, and that earlier research has shown that it is unrealistic to expect that all patients complete an entire intervention.^20^

### Intervention

#### Internet-based intervention “Dealing with Aggression”

In this study, the existing internet-based intervention “Dealing with Aggression” is evaluated as an addition to in-person TAU. This intervention is part of the eHealth-platform Minddistrict. Minddistrict is a platform that offers over 300 different internet-based interventions and is used by over 250,000 people in Europe. Minddistrict is primarily targeted at mental healthcare and contains a broad range of interventions, focused on, for example, mindfulness, autism, healthy lifestyle, and depression. All interventions of Minddistrict, including “Dealing with Aggression,” are developed via the “Intervention Mapping” approach.^32^ Intervention Mapping is a systematic approach which uses theory and evidence as foundations for intervening in health problems. Interventions developed via Intervention Mapping contain behavior change techniques (BCTs), which are specific components designed to change behavior, such as goal-setting or feedback.^33^ Furthermore, by incorporating persuasive features such as personalization, reminders, or rewards, the chances of users being adherent to the intervention and consequently changing their attitudes and behavior change increase.^34^ Finally, therapists are actively involved in the development of interventions to ensure a good fit with the needs and knowledge from clinical practice.

The internet-based intervention “Dealing with Aggression” was first introduced in the Netherlands over 10 years ago, and was updated in 2022. It's effectiveness has not been studied yet. This intervention has to be used in a blended way, that is, integrated in face-to-face treatment. In this intervention, patients learn to deal with conflict and triggers in a constructive way. The intervention is focused on three main objectives: (1) increasing the motivation to change, (2) acquiring skills for dealing with conflict, and (3) breaking the cycle of aggression by providing knowledge on situational, emotional, cognitive, and physical triggers. The module consists of 10 lessons: (1) Motivation: Where are you now? (2) Where do you want to go? (3) Circle of Aggression, (4) Thoughts, (5) Emotions, (6) Bodily sensations, (7) Techniques for self-control, (8) Asking for help, (9) Assertiveness, and (10) Relapse prevention. In Supplemental Appendix A, a more elaborate description of the content of each lesson is provided, and screenshots can be found in Supplemental Appendix B. Each lesson consists of multiple components, that is, written text, assignments, and explanatory videos with therapists and/or experts by experience. When a patient completes a lesson, the therapist has to send written feedback on the assignments. Because “Dealing with Aggression” is used in a blended way, each lesson should be briefly discussed in an in-person treatment session. This means that generally, participants work on one lesson per week outside of their treatment sessions.

#### Control group

The control group will receive TAU on aggression regulation, which refers to psychotherapy delivered by a licenced psychologist to an outpatient. Because this is a multicenter study, there are minor differences between TAU for aggression between participating clinics. To illustrate, some clinics use the “AR op Maat” [Aggression regulation—tailored] program, while others use “Grip op Agressie” [Getting a hold on aggression]. While there are minor differences in the way of delivery of these types of treatment, they are all based on the same underlying principles and models. All treatment programs in forensic psychiatry targets dynamic risk factors that have been identified by means of risk assessment instruments, based on the Risk-Need-Responsivity (RNR) model.^35^ In other words: at the beginning of treatment, an overview of individually relevant risk factors that predict recidivism is always generated by means of risk assessment instruments. These risk factors, for example, impulsiveness or addiction, are then used as the foundation for treatment and the selection for specific interventions. Treatment is shaped by elements from CBT, often combined with techniques from occupational therapy. Furthermore, because of the heterogeneity of the forensic population, there often is variation in the length of treatment and the exact way it is delivered, even when following the same protocol. This is done to ensure that treatment is tailored—in forensic terms, responsive.^35^ Consequently, it is unfeasible and undesirable to offer exactly the same TAU to each patient. No major differences in outcomes between different treatment programs are expected because they are based on the same principles.

### Materials

An overview of the used materials in this study is provided in Table 1. The number of self-report questionnaires and the required invested time is kept as short as possible to increase feasibility. Since no previous RCTs on internet-based interventions in forensic outpatient care were identified, outcome measures were based on qualitative research on experienced benefits of internet-based interventions in forensic psychiatry.^20,25^

**Table 1.** Overview of assessment instruments, measurement moments, and estimated invested time for participants.

Variable	Instrument	T0	T1	T2	T3	Invested time
Aggression	Aggression Questionnaire^36^	X	X	X	X	5 minutes, 20 minutes in total
Regulatory emotional self-efficacy	Regulatory Emotional Self-efficacy (RESE) scale^37^	X	X	X	X	5 minutes, 20 minutes in total
Treatment readiness	Corrections Victoria Treatment Readiness Questionnaire (CVTRQ)^38^	X	X	X	X	5 minutes, 20 minutes in total
Risk assessment	Forensisch Ambulante Risico Evaluatie (FARE)^39^	X			X	Part of standard procedures
Engagement	TWente Engagement with Ehealth and Technologies Scale (TWEETS)^40^	X	X	X		5 minutes, 15 minutes in total (only in experimental group)
Adherence	Log data	X	X	X		Automatically logged
patient experiences	Semistructured interviews			X		Ca. 30 minutes
Therapists experiences	Semistructured interviews				X	Ca. 30 minutes

#### Primary outcome measure

##### Aggression

Because this is the first RCT on internet-based interventions in forensic outpatient care, no previous research can be used to identify the most suitable primary outcome measure. However, since the focal point of the studied internet-based intervention lies on aggression, this construct is selected. Self-reported aggression is assessed by means of the Aggression Questionnaire (Aangepaste Versie van de Agressie Vragenlijst; AVL-AV).^36,41^ The 12 items of the questionnaire are divided into four subscales: physical aggression, verbal aggression, anger, and hostility. The AVL-AV had been shown to be reliable and valid in earlier research.^36^

#### Secondary outcome measures and predictors

##### Regulatory emotional self-efficacy

Because of the content and design of an internet-based intervention, and because forensic psychiatric patients can work part of their treatment independently, their confidence in regulating their emotions might increase.^20^ Consequently, an outcome measure of this study is regulatory emotional self-efficacy, measured by means of the 12-item Regulatory Emotional Self-efficacy (RESE) scale.^37^ The RESE scale assesses self-efficacy in managing negative emotions (8 items) and in expressing positive emotions (4 items). Negative emotional self-efficacy refers to the capability to ameliorate negative emotional states and avoid being overcome by negative emotions such as anger or irritation. Positive self-efficacy is the perceived capability to express positive emotions such as joy or pride. Earlier research has demonstrated the validity and reliability of the RESE scale.^37,42^

##### Treatment readiness

Because internet-based interventions might be aligned with skills and preferences of the patient, the fit between treatment and their preferences might increase.^19^ This can result in higher treatment readiness, which refers to the presence of factors that contribute to engagement and therapeutic change. Treatment readiness is assessed by the Corrections Victoria Treatment Readiness Questionnaire (CVTRQ): a self-report questionnaire that was validated in earlier research.^38^ The total scale consists of 20 items, divided into 4 subscales. The subscale Attitude and Motivation (AM) measures attitudes and beliefs about treatment programs and the desire to change, emotional reactions (ER) measures emotional responses to the individual's offending behavior, offending beliefs (OB) refers to beliefs about personal responsibility for offending behavior, and efficacy (EF) measures perceived ability to participate in treatment programs.

##### Treatment duration

In order to investigate if the use of internet-based intervention leads to a need for less treatment sessions the number of sessions for each participating patient during the data collection period is counted. This is done by asking the responsible therapists for the number of sessions during the period of data collection by means of interviews.

##### Dynamic risk factors

In treatment of forensic psychiatric patients, risk assessment has to be conducted by means of evidence-based risk assessment instruments. In forensic psychiatric outpatient care, the Dutch standard is the Forensisch Ambulante Risico Evaluatie (FARE), version 2.^39^ Research into inter-reliability and convergent validity has shown promising results.^43^ According to Dutch guidelines, the FARE has to be administered once every 6 months. Ideally, the use of internet-based interventions results in increased improvement of dynamic risk factors that are relevant for a patient. In the FARE, 6 unchangeable, static, and 11 changeable, dynamic risk factors are assessed. In this study, only changeable dynamic risk factors are assessed^44^: malfunctioning on education/work, financial mismanagement, delinquent social network, limited leisure activities, problematic (former) partner relationship, instable living situation, problematic substance use, limited impulse control, malfunctional coping skills, antisocial attitude, and rule-violating behavior.^39^

##### Adherence

Adherence is an important construct in explaining why internet-based interventions are (in)effective.^29^ Multiple studies indicate a dose–response relationship, in which there is a positive relationship between the number of completed lessons and the effectiveness.^45^ However, patients might also stop using the intervention prematurely because they feel they have optimally benefited from it.^29^ To gain more insight into the role of adherence, the number of completed lessons is used as a predictor for effectiveness. This construct is measured via log data on the number of lessons completed by each patient.^46^ These data are collected automatically by the Minddistrict-system.

##### Engagement

Engagement is a nonspecific factor that can be used to predict effectiveness of internet-based interventions. It refers to the extent to which someone is involved or occupied with something.^28^ In this study, engagement is measured with the TWente Engagement with Ehealth and Technologies Scale (TWEETS).^40^ The scale employs a definition of engagement that incorporates behavior, cognition, and affect, and has been shown to have a good validity and reliability.^40^ The TWEETS contains nine items and has three slightly different versions: one for expected engagement—to be used when someone starts using an intervention, one for current engagement—to be used during the use of an intervention, and one for past engagement—to be used when a user has completed or stopped using an intervention.

##### Patient characteristics

To describe the characteristics of the sample, information about treatment objectives, age, gender, diagnoses, and the inpatient clinic in which the patient is treated will be collected. These data will be retrieved at T0 from the therapist by means of an online questionnaire. In this way, the researchers do not need access to the personal electronic patient file to protect patient privacy.

#### Qualitative data

##### Patient interviews

A randomly selected sample of 15 patients of the experimental condition, generated by means of a website (https://sealedenvelope.com/), is invited to participate in a postintervention interview. Because the perspective of patients who dropped out of the intervention also is relevant, the target group of this 30-minute interview will consist of patients who completed at least one session. A semistructured interview scheme will be used to identify perceived benefits, barriers, and points of improvement of the internet-based intervention. These results will be used to explain the outcomes of the RCT, to further improve the content, design and implementation of the intervention, and to determine whether the selected outcome measures were suitable, or if other measures need to be taken into account in future research.

##### Therapist interviews

At the end of the data collection period, all therapists that participated in the study will be invited for a 30-minute interview. It is expected that approximately 15 therapists will participate in these interviews, which will cover the same topics as the patient interviews.

### Procedure

#### Participant flow

**Figure 1.**
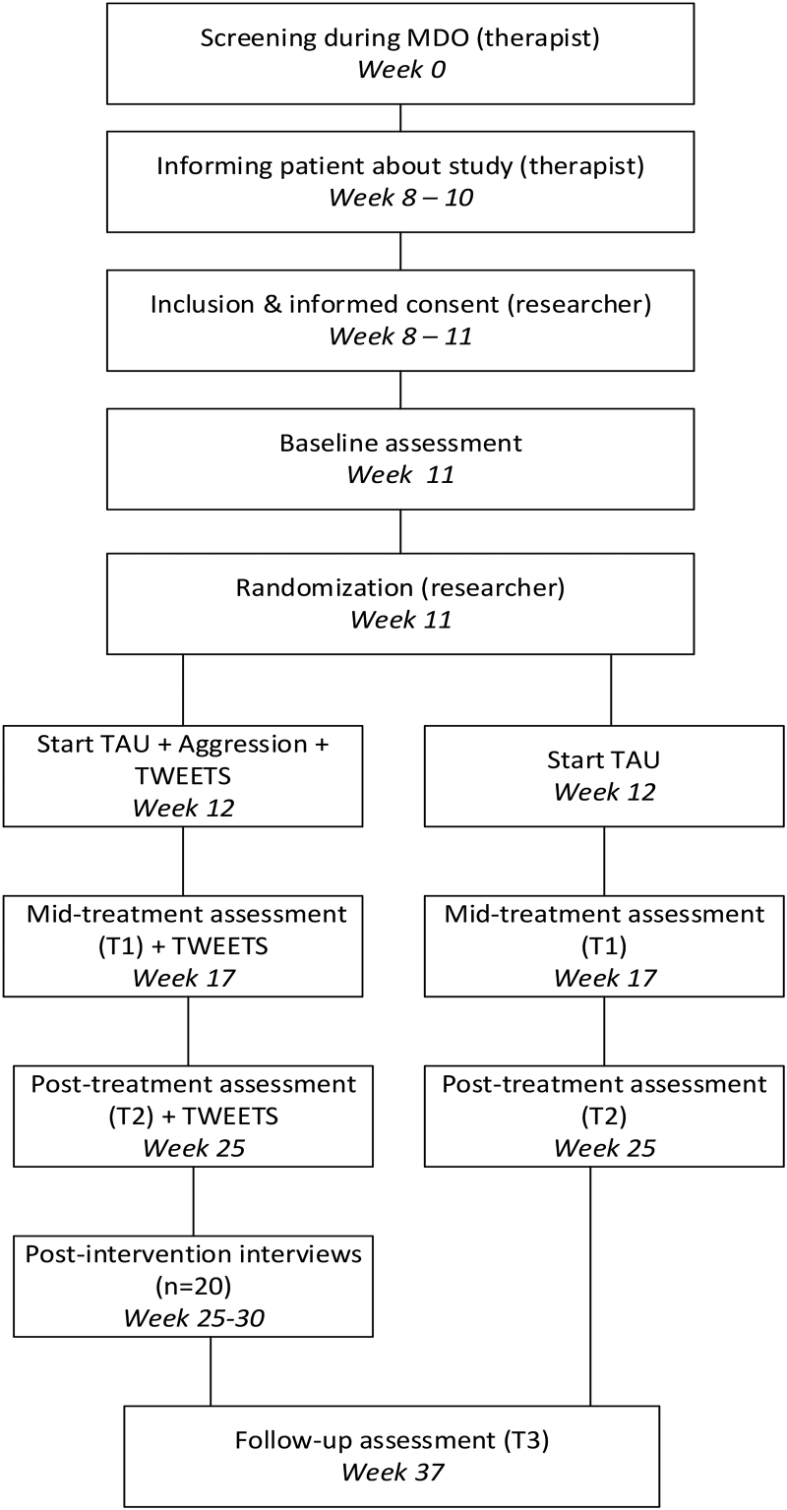
Participant flow through the mixed-methods randomized computed tomography of the internet-based intervention “Dealing with Aggression.”

The flow of the participants throughout the RCT is visualized in Figure 1. A week after patients sign the informed consent form, they receive an email with a link to the questionnaires of the baseline assessment (T0) and then are randomized to the experimental or control condition. Patients will start with the intervention after a maximum of 12 weeks after start of treatment, which can be extended to 14 weeks if necessary. Participants are assessed four times: at baseline (T0), at mid-treatment assessment (T1) when they are supposed to be halfway throughout the internet-based intervention, that is, 6 weeks after baseline; after completing the intervention (T2)—14 weeks after baseline to account for often-occurring delays in treatment; and at follow up (T3), 3 months after completing the intervention. Participants in both conditions will be compensated for their invested time with two €10 vouchers at T1 and T3.

#### Randomization

This study is a nonblinded RCT because of the addition of an intervention to treatment. After screening and informed consent, patients will be randomized by the principal investigator via https://sealedenvelope.com/. Once a patient is included, they receive a number, which will be aligned with a random sequence of treatment allocations, that is, participants are either allocated to the intervention or control group. The first participant included in the study is allocated according to the first treatment allocation on the list, the second participant according to the second allocation, and so forth.

### Statistical analyses

Data will be analyzed using IBM SPSS software (version 24.0) on an intention to treat (ITT) basis. Significance is accepted at 0.05 or lower. Effect sizes and confidence intervals will be used to interpret results.

To determine whether there is more improvement on aggression, regulatory emotional, and treatment readiness in the experimental than the control group during, directly and 3 months after the internet-based intervention “Dealing with Aggression,” multilevel repeated-measures linear mixed models will be used. This method accounts for autocorrelation within participants and missing data. Time and group will be used as fixed factors, along with their interactions, and participants are modeled as random factor. Interaction effects will be studied to identify whether the changes over time differed between groups. Main effects for time will be used to investigate whether the scores of all participants changed over time. In order to provide more insight into the main findings, least-significant difference (LSD) post hoc independent t-tests with Bonferroni correction will be performed. Linear mixed models will also be used to determine if there is more improvement on dynamic risk factors in patients in the experimental condition. To account for potential nonlinear effects, polynomial terms will be included in the regression model. Specifically, quadratic terms for continuous variables will be added such as age and treatment duration. The significance of these polynomial terms will be assessed to determine whether nonlinear relationships were present in our data.

To determine if the number of in-person treatment contacts over a period of 37 weeks is lower in the experimental group, a t-test with group as the independent and number of treatment sessions as dependent variable will be conducted. To investigate whether adherence and engagement predict effectiveness, simple linear regression analyses will be run with the three outcome measures. The score on the TWEETS on T0, T1, and T2 and the number of completed lessons will be used as independent variables, and the change scores—that is, difference between scores on T2 and T0, and difference between scores on T3 and T0—for all outcome measures will be used as dependent variables.

Data of the interviews with patients and therapists will be analyzed via an inductive approach, using the method of constant comparison. First, relevant fragments will be extracted from the anonymized transcripts. Based on these fragments, two researchers will create an initial version of a coding scheme, which will be applied to five interviews, after which it will be constantly adapted using an iterative approach.

After analysis, quantitative and qualitative data will be triangulated in an explanatory mixed-methods design, in which qualitative results are used to illustrate the findings of quantitative analyses.^47^

Because of the multiplicity of the primary outcome measure, multiple scenarios for determining the effectiveness of the intervention can be identified. A first scenario is that no differences between both groups are found on T1, T2, or T3, meaning that the intervention is ineffective in this study. A second scenario is that there is a change on one or two of the outcome measures on T1, T2, and/or T3. This means that the intervention can be viewed as potentially effective because it does result in some improvements, which should be explored in future research. A third scenario is that the improvement on all outcome measures is higher in the experimental group on T3, so the intervention can be viewed as effective.

## Discussion

The main objective of this RCT is to identify whether the addition of the internet-based intervention “Dealing with Aggression” leads to better treatment outcomes for forensic psychiatric outpatients with aggression regulation problems. By using semistructured interviews in an explanatory mixed-methods design, and by adding adherence and engagement as potential predictors, this study can not only answer questions about if, but also why and for whom this intervention works. This first experimental study on an internet-based intervention in a forensic psychiatric outpatient sample will answer an important question from clinical practice: are these types of interventions—which have been used in practice for over 10 years—actually of added value for treatment?

### Implications for practice and research

The outcomes of this study are expected to have multiple implications for clinical practice. If the internet-based intervention proves to be effective, this can provide an impulse for uptake in practice. Research has shown that a lack of evidence is an implementation barrier: therapists and organizations are sometimes hesitant internet-based interventions because it is not clear if, how and for whom they are effective.^20,48,49^ Furthermore, the outcomes of the interviews can provide concrete points of improvements for implementation, highlighting the importance of a mixed-methods approach in eHealth evaluation.^50^ However, if the intervention is ineffective effective, or if—despite elaborate efforts to support participants—very few patients complete the intervention, this also has important implications for implementation. Findings that do not point into the direction of effectiveness should give rise to careful reconsideration of investing resources in these interventions.

This study cannot just provide insight into if, but also why the intervention is (in)effective. In this study, engagement will be analyzed as potential predictor of effectiveness. If engagement at the start predicts effectiveness, it could be possible to identify early on if the intervention will be effective for an individual.^28,40,51^ If engagement is low, this might give rise to changes in delivering the interventions: the patient might require an adapted approach—such as more encouragement by the therapist, or they might benefit more from another type of intervention—such as one focused on mindfulness. Another possibility is that unengaged patients would benefit more from an entirely different type of technology—such as virtual reality or wearables, for which no reading or writing skills are required. By gaining more insight into if an intervention fits with a patient early on in the process, a more adaptive approach that fits the needs and skills of the patients can be created. Information from the interviews will provide additional information on why an intervention does (not) work. Consequently, by integrating different methods, this study will provide answers to questions on why an intervention is effective.

### Limitations and feasibility

While the feasibility of large experimental studies in clinical practice is an issue in almost all settings, it is especially relevant for forensic psychiatry. To account for problems with motivation to participate, the number and length of questionnaires has been kept as short as possible, patients in both groups are compensated for their invested time by means of voucher, patients are frequently reminded to fill out the questionnaires, and a multicenter approach is used to reach as many patients as possible.

A therapist-related limitation is that internet-based interventions are often not introduced at all by therapists, even though they express the intention to do so.^20^ To overcome this “intention-behavior gap,” therapists will be supported and reminded by the researchers to introduce and keep on using intervention to their patients. Furthermore, log data research has shown that there are some “superusers” of internet-based interventions,^20^ so recruitment will be focused on identifying those enthusiastic and experienced therapists.

## Conclusion

This study will provide insight into if and how the internet-based intervention Aggression is of added value for treatment of forensic psychiatric outpatients. If the intervention proves to be effective, this can result in more and better efforts to successfully implement it in clinical practice. On the other hand, if this study shows no evidence for effectiveness, this can give rise to a discussion on if, how, and for whom to use these interventions. In any case, this first mixed-methods RCT on internet-based interventions in forensic outpatient care aims to answer questions that have been asked by practitioners for over 10 years: are these types of interventions of added value for treatment of this complex and hard-to-involve target group?

## Supplemental Material

sj-docx-1-dhj-10.1177_20552076241303835 - Supplemental material for The added value of an internet-based intervention for treatment of aggression in forensic psychiatric outpatients—study protocol for a multicentre, mixed-methods randomized controlled trial Supplemental material, sj-docx-1-dhj-10.1177_20552076241303835 for The added value of an internet-based intervention for treatment of aggression in forensic psychiatric outpatients—study protocol for a multicentre, mixed-methods randomized controlled trial by Hanneke Kip, Lisa Klein Haneveld and Saskia M Kelders in DIGITAL HEALTH
