# Supplementary material for: The added value of an internet-based intervention for treatment of aggression in forensic psychiatric outpatients—study protocol for a multicentre, mixed-methods randomized controlled trial
Source: Digit Health. 2024 Dec 12;10:20552076241303835. doi: 10.1177/20552076241303835 (PMC11639002; doi:10.1177/20552076241303835)

## **Appendix A – A summary of the content of ‘Dealing with Aggression’**

*Table 2*. An overview of the lessons and content of the internet-based intervention ‘Dealing with Aggression’

| **#** | **Lesson** | **Content** |
| --- | --- | --- |
| 1 | Motivation – where are you now? | In this lesson, the patient is introduced to the module, amongst other things by means of a video with a therapist and an audio fragment of an expert by experience. The advantages and disadvantages are discussed by means of open-ended questions. |
| 2 | Where do you want to go? | In this lesson, the patient is asked to think about the future with and without aggression. They are asked about their goals by means of open-ended questions, on which feedback is provided by the therapist. Finally, the patients works on a diary by means of a daily reminders in which they can monitor their own aggressive behaviour and accompanying triggers. |
| 3 | Circle of aggression | In this lesson, the patient is informed about the circle of aggression by means of written text, illustrations and a video. Next, the patient thinks about their own risk situations and accompanying triggers. |
| 4 | Thoughts | In this lesson, the patient receives information about convictions about their past and thinking styles by means of videos, written texts and short assignments. Attention is paid to different perspectives, convictions, thinking errors and the challenging of thoughts. |
| 5 | Emotions | This section starts with information on emotions by means of text and animation. Next, different gradations of emotions are explained, using the metaphor of (over)cooking pans. Via videos, the patient learns about emotions that can lead to aggression. |
| 6 | Bodily sensations | In this lesson, the patient learns more about bodily sensations and risky behaviours that are related to aggression by means of assignments. Additionally, attention is paid to relaxation exercises to deal with bodily sensations. |
| 7 | Techniques for self-control | In this lesson, the patient works on their coping skills and self-efficacy for preventing aggression. The patient leans about potential coping skills such as avoidance, stepping out of the situation and time-outs by means of text and videos with experts by experience. |
| 8 | Asking for help | In this lesson, the patient learns how to ask for help from others and gains insight via text and video on how to deal with others. Furthermore, the patient is encouraged to practice with these coping strategies in real life. |
| 9 | Assertiveness | In this lesson, the patient gains knowledge about reacting in sub-assertive, assertive and aggressive ways. By means of quizzes, text and videos, the patient learns about and can practice with coping skills to deal with aggression. |
| 10 | Relapse prevention | In the final lesson, the patient learns about relapse prevention plans via texts and videos with experts by experience. The patient creates their own relapse prevention plan and makes plans for their future. |

## **Appendix B – Screenshots of the Dutch internet-based intervention ‘Dealing with Aggression’**

*
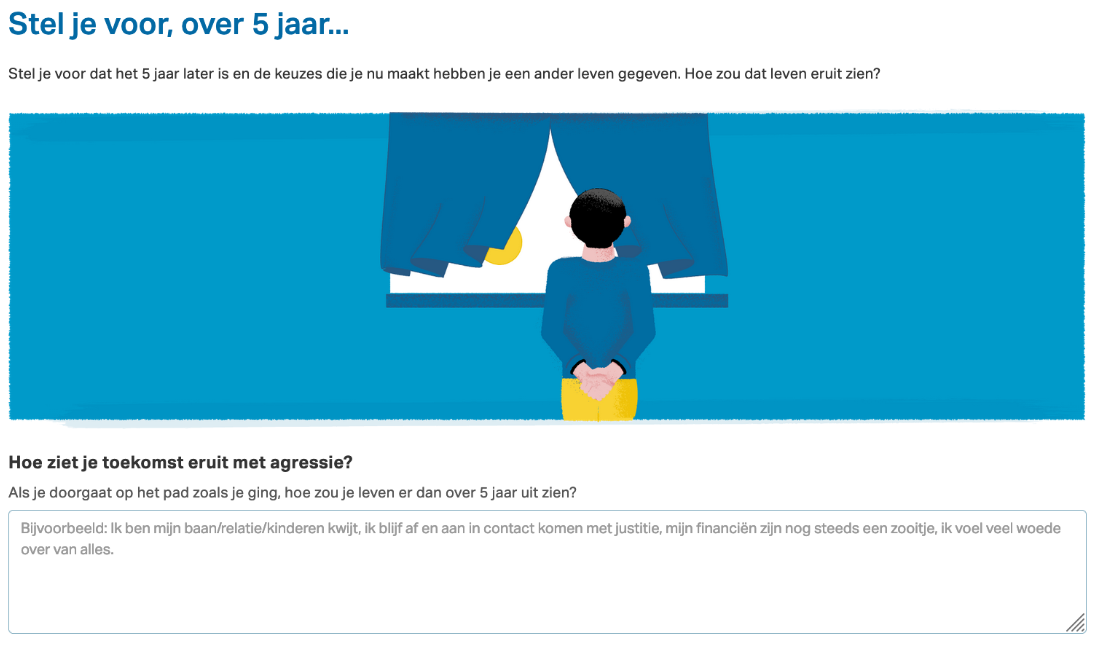
Figure 2.* Screenshot of a written assignment on what the patient’s future will look like in five years with aggression.

*Figure 3.* Screenshot of a short written text and accompanying video on the role that one’s thoughts play in the circle of violence.


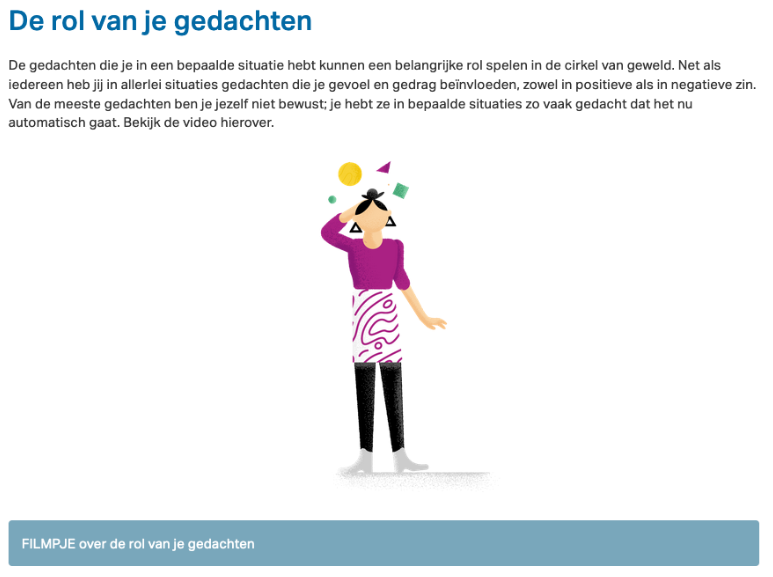


*Figure 4.* Screenshot of a visualization of the ‘circle of violence’


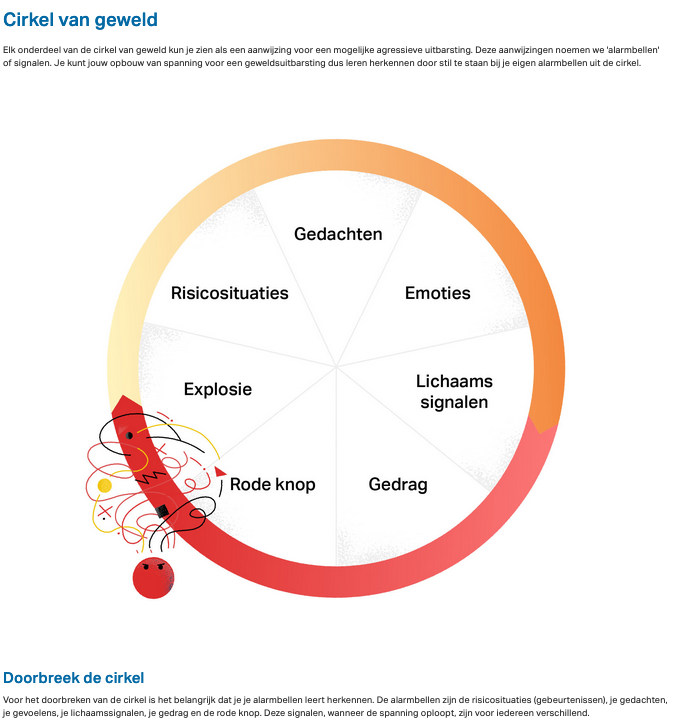

Supplement: sj-docx-1-dhj-10.1177_20552076241303835 - Supplemental material for The added value of an internet-based intervention for treatment of aggression in forensic psychiatric outpatients—study protocol for a multicentre, mixed-methods randomized controlled trial [file sj-docx-1-dhj-10.1177_20552076241303835.docx]
